# Characteristics of Patients with Spontaneous Coronary Artery Dissection Presenting with Sudden Cardiac Arrest in the United States and the Potential Role of Implantable Cardioverter Defibrillator Therapy

**DOI:** 10.31083/j.rcm2509318

**Published:** 2024-09-09

**Authors:** Chayakrit Krittanawong, Yusuf Kamran Qadeer, Song Peng Ang, Zhen Wang, Mahboob Alam, Samin Sharma, Hani Jneid

**Affiliations:** ^1^Cardiology Division, NYU Langone Health and NYU School of Medicine, New York, NY 10016, USA; ^2^Division of Cardiology, Department of Medicine, Henry Ford Hospital, Detroit, MI 48202, USA; ^3^Division of Internal Medicine, Rutgers Health Community Medical Center, Newark, NJ 08903, USA; ^4^Robert D. and Patricia E. Kern Center for the Science of Health Care Delivery, Mayo Clinic, Rochester, MN 55903, USA; ^5^Division of Health Care Policy and Research, Department of Health Sciences Research, Mayo Clinic, Rochester, MN 55903, USA; ^6^The Texas Heart Institute, Baylor College of Medicine, Houston, TX 77030, USA; ^7^Cardiac Catheterization Laboratory of the Cardiovascular Institute, Mount Sinai Hospital, New York, NY 10018, USA; ^8^John Sealy Distinguished Centennial Chair in Cardiology, Chief, Division of Cardiology, University of Texas Medical Branch, Houston, TX 77058-3609, USA

**Keywords:** spontaneous coronary artery dissection, SCAD, cardiac arrest: ICD, ventricular arrhythmia

## Abstract

**Background:**

Spontaneous coronary artery dissection (SCAD) is a disease entity that often occurs in young, healthy women and can cause life-threatening ventricular arrhythmias and sudden cardiac arrest. However, the characteristics and outcomes of SCAD with cardiac arrest are not well characterized.

**Methods:**

This study investigated the baseline characteristics of SCAD patients with cardiac arrest using the National Inpatient Sample (NIS) database between 2016 and 2020. In addition, we also sought to determine the potential impact that implantable cardioverter defibrillator (ICD) therapy had on morbidity and mortality in SCAD patients presenting with cardiac arrest.

**Results:**

Our findings showed that the SCAD with cardiac arrest population had significantly higher comorbidities, including cardiac arrhythmias, congestive heart failure, pulmonary circulation disorders, liver diseases, solid tumors, coagulopathy, fluid disorders, chronic kidney disease (CKD), anemia secondary to deficiency, psychosis, neurological disorders, carotid artery disease, atrial fibrillation, ventricular arrhythmias (ventricular tachycardia (VT), ventricular fibrillation (VF)), and acute myocardial infarction (AMI), compared to the SCAD without cardiac arrest population. Likewise, for SCAD patients who did not have an ICD in place, we found increasing age, fluid and electrolyte disorders, uncomplicated diabetes, neurological disorders, peripheral vascular disease, pulmonary circulatory disorders, cardiac arrhythmias, and congestive heart failure to be associated with greater mortality.

**Conclusions:**

SCAD patients with certain comorbidities (e.g., pulmonary diseases, liver diseases, cancers, coagulopathy, and CKD) who presented with AMI or congestive heart failure should be monitored closely for ventricular arrhythmias as they have a higher chance of progressing to cardiac arrest. ICD therapy can be considered for these patients, but data on the success of this treatment option are limited, and more research needs to be performed to determine whether the benefits of this outweigh the risks.

## 1. Introduction

Spontaneous coronary artery dissection (SCAD) is a heterogeneous condition that normally occurs in young, otherwise healthy women. Moreover, SCAD has received more attention recently as this disease process can cause life-threatening ventricular arrhythmias and cardiac arrest. A recent review article by Kaddoura and colleagues provided a comprehensive overview of this disease process. The authors discussed how the pathogenesis of SCAD is most likely secondary to an intimal tear of the epicardial coronary arteries, leading to intramural hematoma formation and subsequent occlusion of the epicardial lumen [1, 2, 3]. Patients with SCAD typically present with symptoms of acute coronary syndrome [4]. Diagnosis is usually made via coronary angiogram, along with intravascular ultrasound and optical coherence tomography being used as adjunct modalities [5]. Based on international guidelines, treatment generally favors early revascularization over medical therapy in unstable patients [6]. However, the characteristics and outcomes of SCAD with cardiac arrest are not yet well characterized. Likewise, the benefit of an implantable cardioverter defibrillator (ICD) in SCAD patients with cardiac arrest is unknown. This study investigated baseline characteristics in SCAD patients with cardiac arrest using the National Inpatient Sample (NIS) database between 2016 and 2020. We also evaluated the outcomes and their predictors in SCAD–cardiac arrest with or without an ICD.

## 2. Methods

### 2.1 Data Source

We queried the NIS database from 2016 to 2020. The NIS is the largest publicly available all-payer inpatient healthcare database that could produce U.S. regional and national estimates of inpatient utilization, access, cost, quality, and outcomes at both regional and national levels in the U.S. Its unweighted form includes information from approximately 7 million annual hospital stays. When weighted, it projects an estimation of about 35 million hospitalizations across the nation each year. The NIS encompasses data from states involved in the Healthcare Cost and Utilization Project (HCUP), representing over 97% of the United States population. It effectively approximates a 20% stratified sample of patient discharges from U.S. hospitals, excluding facilities specializing in rehabilitation and long-term acute care. In addition, given that the data contained within NIS are deidentified, our study did not require approval from the Institutional Review Board.

### 2.2 Study Population

From 2016 to 2020, the NIS contained up to 40 diagnoses and 25 procedures for each admission. Patients with a primary or secondary SCAD diagnosis were identified using the ICD-10-CM code I25.42. To ensure an appropriate diagnosis of SCAD, we selected patients who presented with acute myocardial infarction (AMI), defined as either non-ST-segment elevation or ST-segment elevation. Following that, we selected only patients with a procedural diagnosis of coronary angiography or percutaneous coronary intervention (PCI) and excluded any patients with concurrent diagnoses of accidental puncture or laceration to decrease the risk of coding errors. All adult hospitalizations (>18 years of age) were included in our study. Hospitalization with missing data on age, gender, race, mortality, type of admission (elective vs. non-elective), median household income, and primary payment coverage were excluded from our study. Our methodology aligned with previously published literature and standards recommended by the Agency of Healthcare Research and Quality [7].

### 2.3 Study Covariates and Outcomes 

To ensure a robust analysis and to minimize unrecognized confounders, we included a large number of covariates. Data on demographics (age, gender, insurance status, hospital bed size, hospital teaching status, elective admission, and race) were readily available within the database. Additional comorbidities included obesity, hypertension, ventricular arrhythmias, valvular heart diseases, pulmonary circulatory disorders, chronic lung diseases, liver diseases, diabetes mellitus, peripheral vascular diseases, lymphoma, metastatic cancer, solid tumors, rheumatological disorders, coagulopathy, fluid disorders, chronic kidney disease, blood loss anemia, deficiency anemia, alcohol abuse, drug abuse, acquired immunodeficiency syndrome (AIDS), prior myocardial infarction (MI), prior PCI, and prior coronary artery bypass graft (CABG), which were extracted according to their respective ICD-10-CM codes. Ventricular arrhythmias are defined as a composite of ventricular tachycardia or ventricular fibrillation. The category ‘other neurological disorders’ includes a range of neurological conditions, including ataxia, spastic paraplegia, spinocerebellar disease, chorea, multiple sclerosis, demyelinating diseases, epilepsy, seizures, convulsions, aphasia, Parkinson’s disease, neuroleptic malignant syndrome, and various other degenerative brain diseases not classified elsewhere.

Our study objective was to evaluate the demographics, clinical characteristics, and outcomes of SCAD patients stratified by the sudden cardiac arrest and ICD, respectively. Secondary outcomes included exploring the predictors of cardiac arrest and the ICD among patients with SCAD, as well as evaluating the predictors of mortality among patients who did not receive an ICD. We additionally assessed the temporal trend of incidence of sudden cardiac arrest among SCAD patients and in-hospital mortality among sudden cardiac arrest patients.

### 2.4 Statistical Analysis

The national weighted estimates were obtained using the discharge weight supplied by HCUP. Categorical variables were presented as counts and percentages and analyzed using the chi-square test. Alternatively, continuous variables were described using weighted means and standard deviations for those following a normal distribution or medians and interquartile ranges for those not normally distributed. Trends of sudden cardiac arrest and in-hospital mortality were summarized and analyzed using ogistic regression. We first obtained the absolute frequencies of each desired outcome, comparing those with and without cardiac arrest and the ICD, respectively. We rigorously adhered to the data use agreement for nationwide databases from the HCUP and avoided reporting any variable with a frequency of 10 and below, which was excluded given the risk of identifying individual patients [8]. We conducted a multivariate regression analysis using the multilevel mixed effect models to evaluate for predictors of cardiac arrest, ICD placement among SCAD patients, and predictors of mortality among patients who did not receive an ICD. Variables included in the model were any significant variables on univariate analysis using a liberal threshold of 0.2. All statistical analyses were conducted using Stata version 17.0 (StataCorp, College Station, TX, USA) and R software version 4.3 (R Foundation for Statistical Computing, Vienna, Austria).

## 3. Results

We analyzed 24,620 patients with spontaneous coronary artery dissection, 1125 of which suffered cardiac arrest and 23,495 who did not suffer cardiac arrest. Of the patients with SCAD who also suffered a cardiac arrest, the mean age was 62.4 years, with 57% of the patients being female and most patients being Caucasian. In comparison, of the patients with SCAD who did not have a cardiac arrest, the mean age was 59.8 years, with 59.6% of the patients being female and most patients being Caucasian. The SCAD with cardiac arrest population had significantly higher comorbidities, including ventricular arrhythmias, congestive heart failure, pulmonary circulatory disorders, liver diseases, solid tumors, coagulopathy, obesity, fluid disorders, chronic kidney disease (CKD), psychosis, and neurologic disorders, compared to the SCAD without cardiac arrest population (Table 1).

**Table 1.** **Baseline characteristics of SCAD patients with and without cardiac arrest**.

Variables		No cardiac arrest population		Cardiac arrest population		*p*-value
Number of patients		23,495		1125		
Age		59.83 ± 14.36		62.35 ± 15.29		0.02
		n	%	n	%	
Female		14,005	59.6	645	57.3	0.50
Race						0.34
	White	17,435	74.2	830	73.8	
	Black	2700	11.5	135	12.0	
	Hispanic	2090	8.9	85	7.6	
	Asian or Pacific Islander	520	2.2	15	1.3	
	Native American	110	0.5	15	1.3	
	Other	640	2.7	45	4.0	
Hospital bed size						0.55
	Small	3295	14.0	185	16.4	
	Medium	6615	28.2	295	26.2	
	Large	13,585	57.8	645	57.3	
Hospital teaching status						0.25
	Rural	1100	4.7	45	4.0	
	Urban non-teaching	4130	17.6	245	21.8	
	Urban teaching	18,265	77.7	835	74.2	
Admission						
	Elective	1885	8.0	90	8.0	0.99
Primary payment coverage						0.38
	Medicare	9130	38.9	515	45.8	
	Medicaid	2530	10.8	115	10.2	
	Private insurance	9870	42.0	420	37.3	
	Self-pay	1170	5.0	50	4.4	
	No charge	-	-	-	-	-
	Other	685	2.9	25	2.2	
Median household income						0.50
	1–28,999	5990	25.5	315	28.0	
	29,000–35,999	5890	25.1	235	20.9	
	36,000–46,999	6495	27.6	310	27.6	
	47,000+	5120	21.8	265	23.6	
Hospital region						0.06
	Northeast	4250	18.1	155	13.8	
	Midwest	5485	23.3	340	30.2	
	South	8755	37.3	420	37.3	
	West	5005	21.3	210	18.7	
Comorbidities						
	Congestive heart failure	7245	30.8	575	51.1	<0.001
	Ventricular arrhythmias	2955	12.6	760	67.6	<0.001
	Valvular heart diseases	2505	10.7	135	12.0	0.52
	Pulmonary circulatory disorders	780	3.3	85	7.6	<0.001
	Peripheral vascular disease	2585	11.0	140	12.4	0.50
	Hypertension	16,450	70.0	4303	67.6	0.43
	Other neurologic disorders	1385	5.9	280	24.9	<0.001
	Chronic lung disease	4130	17.6	240	21.3	0.15
	Diabetes mellitus	5610	23.9	325	28.9	0.08
	Hypothyroidism	2705	11.5	90	8.0	0.11
	CKD	2845	12.1	215	19.1	0.00
	Liver disease	1025	4.4	120	10.7	<0.001
	Solid tumor	335	1.4	40	3.6	0.01
	Rheumatologic disorders	550	2.3	45	4.0	0.11
	Coagulopathy	1635	7.0	200	17.8	<0.001
	Obesity	5135	21.9	175	15.6	0.03
	Weight loss	575	2.4	30	2.7	0.84
	Fluid and electrolyte disorders	5225	22.2	530	47.1	<0.001
	Anemia	875	3.7	40	3.6	0.90
	Alcohol abuse	595	2.5	45	4.0	0.18
	Drug abuse	955	4.1	45	4.0	0.96
	Depression	2510	10.7	120	10.7	0.99
	Prior MI	2855	12.2	120	10.7	0.50
	Prior CABG	980	4.2	40	3.6	0.65
Mortality		310	1.3	1030	91.6	<0.001
Cost of hospitalization		31,436 ± 35,012		52,922 ± 47,636		<0.001
LOS		4.68 ± 6.05		8.36 ± 10.57		<0.001

CABG, coronary artery bypass graft; CKD, chronic kidney disease; LOS, length of stay; MI, myocardial infarction; SCAD, spontaneous coronary artery dissection. Any variable with ≤10 patients was not reported per Agency for Healthcare Research and Quality (AHRQ) guidelines and appears as (-) in the table.

**Fig. 1.**
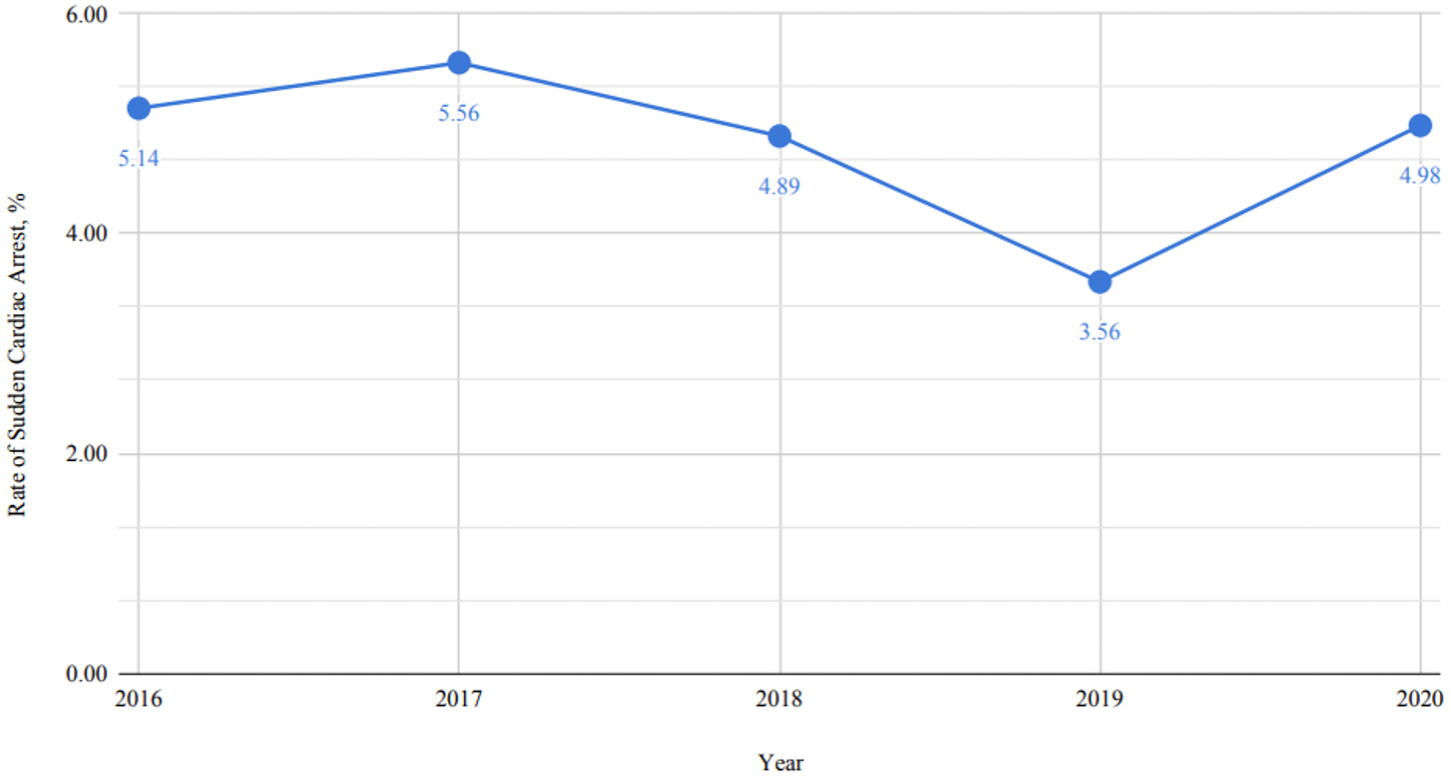
**Temporal trends of cardiac arrest among patients with spontaneous coronary artery dissection (SCAD)**.

**Fig. 2.**
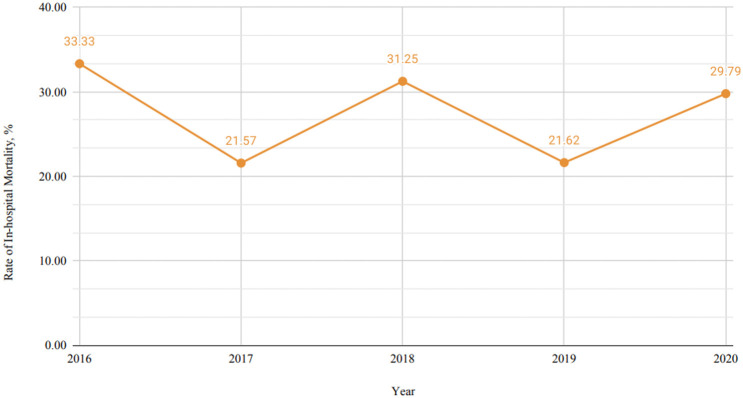
**Temporal trend of in-hospital mortality among spontaneous coronary artery dissection (SCAD) patients with cardiac arrest**.

In addition, we constructed a model to assess which comorbidities were associated with a greater risk of cardiac arrest (Table 2), which according to our model were ventricular arrhythmias (OR 12.11 with 95% CI (8.78–16.70) *p*-value < 0.0001), fluid and electrolyte disorders (OR 1.42 with 95% CI (1.01–2.01) *p*-value 0.045), and other neurological disorders (OR 2.55 with 95% CI (1.69–3.85) *p*-value < 0.0001). Fig. 1 shows the temporal trend of sudden cardiac arrest in SCAD patients from 2016 to 2020. The rate of sudden cardiac arrest peaked in 2017 at 5.56%, then trended downwards over the next two years, with a low of 3.56% in 2019 before rising to 4.98% in 2020. Fig. 2 shows the temporal trend of in-hospital mortality among patients with SCAD who experienced sudden cardiac arrest from 2016 to 2019. There was high variability, as the in-hospital mortality in 2016 was 33.33% in 2016, 21.57% in 2017, 31.25% in 2018, 21.62% in 2019, and 29.79% in 2020. The trends of sudden cardiac arrest incidence in SCAD patients and in-hospital mortality among sudden cardiac arrest patients were analyzed; however, neither showed statistically significant changes over time (*p*-trend = 0.317 and 0.838, respectively).

**Table 2.** **Multivariate logistic regression for cardiac arrest prediction**.

Variables		Odds ratio	95% CI		*p*-value
			Lower limit	Upper limit	
Age		1.01	0.99	1.02	0.36
Hospital region					
	Northeast	Ref			
	Midwest	1.58	0.98	2.56	0.06
	South	1.24	0.77	2.00	0.38
	West	1.00	0.60	1.66	0.99
Congestive heart failure		1.17	0.84	1.63	0.35
Ventricular arrhythmias		12.11	8.78	16.70	< **0.001**
Pulmonary circulatory disorders		1.53	0.84	2.77	0.17
Other neurological disorders		2.55	1.69	3.85	< **0.001**
Chronic lung disease		1.07	0.74	1.55	0.73
Diabetes mellitus		1.41	0.98	2.02	0.06
Hypothyroidism		0.75	0.45	1.25	0.27
CKD		1.09	0.71	1.68	0.69
Liver disease		0.79	0.43	1.43	0.43
Solid tumor		1.99	0.85	4.67	0.11
Rheumatological disorders		2.60	1.19	5.70	**0.02**
Coagulopathy		1.43	0.90	2.27	0.13
Obesity		0.66	0.44	1.01	0.06
Fluid and electrolyte disorders		1.42	1.01	2.01	**0.045**
Alcohol abuse		1.37	0.68	2.77	0.38

CI, confidence interval; CKD, chronic kidney disease. The model was constructed based on univariate regression, with a threshold of 0.2. The bolded *p*-value in Table 2 indicates that cardiac arrest higher in those with ventriuclar arrtyhmias like ventriuclar tachycardia or ventricular fibrillation, neurological disorders (which included disorders like stroke, multiple sclerosis), rheumatological disorders (like lupus), and fluid/electrolyte disorders (like hypokalemia, hyponatremia, etc).

Among 24,620 SCAD patients, 0.6% underwent ICD placement, and 99.4% did not. The mean age was 61.8 years among SCAD patients who received an ICD, while the mean age was 59.9 years among SCAD patients who did not receive an ICD. Compared with SCAD patients without an ICD, those with an ICD were associated with congestive heart failure and cardiac arrhythmias (*p* < 0.05). In addition, compared with SCAD patients without an ICD, those with an ICD had a significantly higher prevalence of pulmonary circulatory disorders, neurologic disorders, liver disease, fluid and electrolyte disorders, and alcohol abuse (*p* < 0.05) (Table 3).

**Table 3.** **Baseline characteristics of SCAD patients with and without ICD placement**.

Variables		No ICD		ICD		*p*-value
Number of patients		24,480		140		
Age		59.93 ± 14.40		61.82 ± 15.00		0.506
		n	%	n	%	
Female		14,580	59.56	70	50.00	0.3041
Race						
	White	18,150	74.14	115	82.14	<0.001
Hospital bed size						0.6797
	Small	3455	14.11	25	17.86	
	Medium	6880	28.10	30	21.43	
	Large	14,145	57.78	85	60.71	
Hospital teaching status						0.3033
	Rural	-	-	-	-	
	Urban non-teaching	-	-	-	-	
	Urban teaching	18,975	77.51	125	89.29	
Primary payment coverage						0.8493
	Medicare	9600	39.22	45	32.14	
	Medicaid	2625	10.72	20	14.29	
	Private insurance	10,225	41.77	65	46.43	
	Self-pay	-	-	-	-	
	No charge	-	-	-	-	
	Other	-	-	-	-	
Median household income						0.7919
	1–28,999	6260	25.57	45	32.14	
	29,000–35,999	6100	24.92	25	17.86	
	36,000–46,999	6765	27.63	40	28.57	
	47,000+	5355	21.88	30	21.43	
Hospital region						0.0148
	Northeast	4350	17.77	55	39.29	
	Midwest	5790	23.65	35	25.00	
	South	9135	37.32	40	28.57	
	West	-	-	-	-	
Congestive heart failure		7710	31.50	110	78.57	<0.001
Ventricular arrhythmias		3590	14.67	125	89.29	<0.001
Valvular heart diseases		2615	10.68	25	17.86	0.2216
Pulmonary circulatory disorders		845	3.45	20	14.29	0.002
Peripheral vascular disease		2705	11.05	20	14.29	0.5865
Hypertension		17,125	69.96	85	60.71	0.2892
Diabetes mellitus		5920	24.18	15	10.71	0.0966
Other neurologic disorders		1640	6.70	25	17.86	0.0191
Chronic lung disease		4355	17.79	15	10.71	0.3287
Hypothyroidism		2770	11.32	25	17.86	0.276
CKD		3045	12.44	15	10.71	0.7824
Liver disease		1110	4.53	35	25.00	<0.001
Obesity		5280	21.57	30	21.43	0.9857
Weight loss		585	2.39	20	14.29	0.0001
Fluid and electrolyte disorders		5680	23.20	75	53.57	0.0001
Alcohol abuse		625	2.55	15	10.71	0.0069
Prior MI		2955	12.07	20	14.29	0.7197
Cost of hospitalization		32,015 ± 35,369		102,137 ± 61,756		<0.001
LOS		4.80 ± 6.30		14.71 ± 9.95		<0.001
Mortality		-	-	-	-	

CKD, chronic kidney disease; LOS, length of stay; MI, myocardial infarction; SCAD, spontaneous coronary artery dissection; ICD, implantable cardioverter defibrillator. *Any variable with ≤10 patients was not reported per Agency for Healthcare Research and Quality (AHRQ) guidelines and appears as (-) in the table. ^+^Data on other races are not reported as this contained a cell count of ≤10.

Similarly, we conducted a model to predict which comorbidities were associated with a greater risk of ICD placement (Table 4), which according to our model were congestive heart failure (OR 3.99 with 95% CI (1.46–10.87) *p*-value 0.01), ventricular arrhythmias (OR 33.94 with 95% CI (9.20–125.18) *p*-value 0.001), pulmonary circulatory disorders (OR 4.40 with 95% CI (1.34–14.44) *p*-value 0.02), and alcohol abuse (OR 4.41 with 95% CI (1.22–15.94) *p*-value 0.02).

**Table 4.** **Multivariate logistic regression for ICD prediction**.

Variables	Odds ratio	95% CI		*p*-value
		Lower limit	Upper limit	
Congestive heart failure	3.99	1.46	10.87	**0.01**
Ventricular arrhythmias	33.94	9.20	125.18	< **0.001**
Pulmonary circulatory disorders	4.40	1.34	14.44	**0.02**
Diabetes mellitus	0.29	0.08	1.02	0.05
Other neurological disorders	0.65	0.22	1.94	0.44
Liver disease	2.39	0.78	7.39	0.13
Weight loss	3.13	0.87	11.33	0.08
Fluid and electrolyte disorders	1.21	0.49	2.97	0.68
Alcohol abuse	4.41	1.22	15.94	**0.02**

CI, confidence interval; ICD, implantable cardioverter defibrillator. The model was constructed based on univariate regression, with a threshold of 0.2. The bolded *p*-value in Table 4 indicates that Ventricular arrhythmias like ventricular tachycardia or ventricular fibrillation, pulmonary circulation disorders like pulmonary embolism, and abusing alcohol (more than 3 drinks a day for men and 2 drinks per women) resulted in a greater likelihood of having an ICD placed.

We further evaluated the predictors of mortality among SCAD patients who did not receive an ICD. Based on our model, increasing age (OR 1.06 with 95% CI (1.04–1.07) *p*-value < 0.001), fluid and electrolyte disorders (OR 2.34 with 95% CI (1.73–3.16) *p*-value < 0.001), diabetes (OR 1.64 with 95% CI (1.21–2.23) *p*-value < 0.001), neurological disorders (OR 2.42 with 95% CI (1.63–3.59) *p*-value < 0.001), peripheral vascular disease (OR 1.48 with 95% CI (1.04–2.10) *p*-value 0.03), pulmonary circulatory disorders (OR 2.01 with 95% CI (1.20–3.39) *p*-value 0.01), ventricular arrhythmias (OR 3.25 with 95% CI (2.36–4.48) *p*-value < 0.001), and congestive heart failure (OR 1.57 with 95% CI (1.18–2.10) *p*-value < 0.001) were associated with increased risk of mortality (Table 5). Notably, given the small sample size (n = 140), we did not evaluate the predictors of mortality among SCAD patients who received an ICD.

**Table 5.** **Multivariate logistic regression for mortality prediction in patients without an ICD**.

Variables		Odds ratio	95% CI		*p*-value
			Lower limit	Upper limit	
Age		1.06	1.04	1.07	<0.001
Female		1.24	0.92	1.66	0.16
Hospital bed size					
	Small	Ref			
	Medium	0.67	0.42	1.09	0.11
	Large	0.90	0.59	1.37	0.63
Hospital region					
	Northeast	Ref			
	Midwest	1.02	0.63	1.64	0.95
	South	1.27	0.83	1.95	0.27
	West	1.39	0.87	2.21	0.17
Elective		1.09	0.69	1.72	0.71
Household median income					
	1–28,999	Ref			
	29,000–35,999	0.68	0.47	0.98	0.04
	36,000–46,999	0.61	0.42	0.89	0.01
	47,000+	0.50	0.33	0.75	<0.001
Diabetes		1.64	1.21	2.23	<0.001
Hypertension		0.79	0.56	1.13	0.21
Ventricular arrhythmia		3.25	2.36	4.48	<0.001
Prior CABG		1.15	0.67	1.96	0.62
Depression		0.70	0.40	1.20	0.19
Fluid and electrolyte disorders		2.34	1.73	3.16	<0.001
Weight loss		1.33	0.71	2.51	0.38
Obesity		0.66	0.44	0.99	0.04
Coagulopathy		1.61	1.08	2.40	0.02
Rheumatological disorders		0.33	0.08	1.38	0.13
Liver disease		3.28	2.08	5.15	<0.001
CKD		1.26	0.90	1.77	0.18
Chronic lung disease		0.79	0.56	1.11	0.17
Other neurological disorders		2.42	1.63	3.59	<0.001
Paralysis		0.58	0.17	1.97	0.38
Peripheral vascular diseases		1.48	1.04	2.10	0.03
Pulmonary circulatory disorders		2.01	1.20	3.39	0.01
Valvular heart diseases		0.66	0.43	1.02	0.06
Congestive heart failure		1.57	1.18	2.10	<0.001

CABG, coronary artery bypass graft; CI, confidence interval; CKD, chronic kidney disease; ICD, implantable cardioverter defibrillator. The model was constructed based on univariate regression, with a threshold of 0.2. ^+^Data on other races are not reported as this contained a cell count of ≤10.

## 4. Discussion

In our study, we found that SCAD patients who suffered a cardiac arrest had higher comorbidities, such as congestive heart failure, pulmonary diseases, liver diseases, cancers, coagulopathy, and CKD, compared to SCAD patients who did not suffer a cardiac arrest. SCAD patients who underwent cardiac arrest were associated with AMI and ventricular arrhythmias (ventricular tachycardia (VT), ventricular fibrillation (VF)). We also found that the trend of cardiac arrest in SCAD patients has continually been trending downward, whereas the in-hospital mortality of these patients has remained quite variable. The decline in trends of cardiac arrest in SCAD patients is perhaps due to increased recognition of SCAD and improvement in medical therapy for SCAD.

One prior study also tried to answer the question of which characteristics are inherently present in SCAD patients with cardiac arrest. Phan and colleagues performed a retrospective cohort analysis of 208 SCAD patients from 2006 to 2016. Of those who suffered cardiac arrest, the investigators concluded that this subset was more likely to have coronary lesions involving the left main or left anterior descending artery (LAD) territory. This could explain the results of our findings, as patients who have ischemic disease are more likely to develop cardiac arrhythmia and heart failure [9, 10, 11]. In other words, in SCAD patients who presented with cardiac arrest, it seems they carried an increased risk of developing cardiac arrest secondary to underlying coronary artery disease. Interestingly, they also found that secondary prevention with an ICD did not significantly benefit this population. This brings into question whether ICD therapy is beneficial in SCAD patients with cardiac arrest. Sharma and colleagues also evaluated 102 patients presenting to Massachusetts General Hospital with SCAD. Comparing those presenting with cardiac arrest to those without cardiac arrest, they found that the cardiac arrest subset was more likely to smoke and present with ST elevation myocardial infarction (STEMI). Although these data are from a very small sample size [12], the findings echo our current findings. Both of these studies seem to stress that SCAD patients presenting with cardiac arrest occurred in the presence of underlying heart disease and comorbidities such as smoking.

Although SCAD patients are likely to be healthy and young, SCAD patients with cardiac arrest tend to have risk factors that would predispose them to cardiac arrest. These factors include coronary artery disease, congestive heart failure, and atrial or ventricular arrhythmia. In our prior study from a single healthcare system, we found that ventricular arrhythmia and atrial fibrillation were independently associated with in-hospital mortality in patients with SCAD [13]. The trend in SCAD patients experiencing cardiac arrest decreased from 2017 to 2019, which could be explained by better detection of SCAD in general. The in-hospital mortality variability for SCAD with sudden cardiac arrest (SCA) is probably a combination of factors, including the expertise of each hospital and physicians in treating SCAD patients.

Our study has certain limitations. Notable limitations include the small number of SCAD patients who received ICD. This potentially explained the relatively wide confidence intervals of certain ICD placement predictors, suggesting that these predictors may be imprecise; thus, the results are inconclusive. Other limitations include the lack of essential laboratory and medication data.

## 5. Conclusions

SCAD patients with certain comorbidities (e.g., pulmonary diseases, liver diseases, cancers, coagulopathy, and CKD) who presented with AMI or congestive heart failure should be monitored closely for ventricular arrhythmias as they have a higher chance of progressing to cardiac arrest. More research needs to be conducted to effectively determine how SCAD patients with cardiac arrest should be treated and managed going forward. ICD therapy can be considered for these patients, but data on the success of this treatment option are limited, and more research needs to be performed to determine whether the benefits of this outweigh the risks.

## Availability of Data and Materials

The datasets used and/or analyzed during the current study are available from the corresponding author on reasonable request.
